# Co-occurrence between C1 esterase inhibitor deficiency and autoimmune disease: a systematic literature review

**DOI:** 10.1186/s13223-020-00437-x

**Published:** 2020-05-27

**Authors:** Donald Levy, Timothy Craig, Paul K. Keith, Girishanthy Krishnarajah, Rachel Beckerman, Subhransu Prusty

**Affiliations:** 1 Division of Basic and Clinical Immunology, University of California, 705 W. La Veta Ave STE 101, Orange, CA 92868 USA; 2 Departments of Medicine and Pediatrics, Penn State University, Hershey, PA USA; 3 Department of Medicine, McMaster University, Hamilton, ON Canada; 4 CSL Behring, King of Prussia, PA USA; 5 Maple Health Group LLC, NY, USA; 6 CSL Behring, Marburg, Germany; 7 Present Address: Seqirus–A CSL Company, Summit, NJ USA

**Keywords:** Autoimmune disease, C4, C1 esterase inhibitor, Celiac disease, Crohn’s disease, Glomerulonephritis, Hereditary angioedema, Lupus, Rheumatoid arthritis, Sjogren’s syndrome, Thyroid, Ulcerative colitis

## Abstract

**Background:**

Hereditary angioedema (HAE) is caused by a SERPING1 gene defect resulting in decreased (Type I) or dysfunctional (Type II) C1 esterase inhibitor (C1-INH). The prevalence of autoimmune diseases (ADs) in patients with HAE appears to be higher than the general population. A systematic literature review was conducted to examine the co-occurrence between HAE and ADs.

**Methods:**

PubMed/EMBASE were searched for English-language reviews, case reports, observational studies, retrospective studies, and randomized controlled trials up to 04/15/2018 (04/15/2015-04/15/2018 for EMBASE) that mentioned patients with HAE Type I or II and comorbid ADs. Non-human or in vitro studies and publications of C1-INH deficiency secondary to lymphoproliferative disorders or angiotensin-converting-enzyme inhibitors were excluded.

**Results:**

Of the 2880 records screened, 76 met the eligibility criteria and 155 individual occurrences of co-occurring HAE and AD were mentioned. The most common ADs were systemic lupus erythematosus (30 mentions), thyroid disease (21 mentions), and glomerulonephritis (16 mentions). When ADs were grouped by MedDRA v21.0 High Level Terms, the most common were: Lupus Erythematosus and Associated Conditions, n = 52; Endocrine Autoimmune Disorders, n = 21; Gastrointestinal Inflammatory Conditions, n = 16; Glomerulonephritis and Nephrotic Syndrome, n = 16; Rheumatoid Arthritis and Associated Conditions, n = 11; Eye, Salivary Gland and Connective Tissue Disorders, n = 10; and Immune and Associated Conditions Not Elsewhere Classified, n = 5.

**Conclusions:**

Based on literature reports, systemic lupus erythematosus is the most common AD co-occurring with HAE Type I and II. Cause and effect for co-occurring HAE and AD has not been clinically established but could be related to lack of sufficient C1-INH function.

## Background

The human C1 esterase inhibitor (C1-INH) binds to proteases involved in the initiation of complement pathways, the kallikrein-kinin system (often referred to as the “contact system”), fibrinolysis, and the coagulation cascade [1]. As a regulatory protein, C1-INH downregulates the production of the vasodilator bradykinin in the contact system [1]. In hereditary angioedema (HAE), genetic defects in the C1-INH gene (Serine Protease Inhibitor Gene 1 present on the Long Arm of Chromosome 11 [11q]: SERPING1) can result in a deficient or dysfunctional protein (HAE Type I and II, respectively [HAE-C1INH]) [2, 3]. Subsequently, extravasation of plasma into cutaneous or mucosal tissues can result in angioedema [4, 5]. Patients with HAE may experience angioedema of the face, extremities, intestines, and genitals, and may also experience life-threatening laryngeal edema. Recommended treatments for acute HAE attacks include plasma-derived C1-INH concentrate, recombinant C1-INH (conestat alfa), the bradykinin B2 receptor antagonist icatibant, and the recombinant kallikrein inhibitor ecallantide [6–9]. Therapies for prophylaxis include intravenous and subcutaneous C1-INH (plasma-derived preferred), a monoclonal antibody against kallikrein (lanadelumab), androgens, and tranexamic acid [6–10].

One of the main functions of C1-INH is regulation of the complement pathway by preventing excessive activation of C4 and C2 via inhibition of the complement proteases C1s in the classical pathway, and mannose-binding lectin-associated serine protease 1/2 (MASP1 and MASP2) in the lectin pathway [1, 11]. C1-INH also appears to regulate the alternative complement pathway via binding of C3b [12]. The deficient function of C1-INH in patients with HAE results in autoactivation of C1s, leading to chronic activation and consumption of C4 and other early components of the complement system, with a corresponding decrease in levels of circulating plasma C4 [13, 14]. C2 levels often decline during acute attacks [13].

Although the complement system is best known for its contribution to the defense from microbial pathogens, it also contributes to protection against the development of autoimmune disease (AD) through multiple mechanisms. These protective mechanisms include promotion of antigen/antibody immune complex clearance, clearance of apoptotic cells that could become a source of autoantigens, and contribution to tolerance to self-antigens [15–17]. Genetic deficiencies in early components of complement (C1, C4, and C2) increase the risk of development of the ADs systemic lupus erythematosus (SLE) and glomerulonephritis (GN), presumably because of a decrease in these protective complement mechanisms [17, 18]. Hereditary C1-INH deficiency also appears to be associated with SLE and GN [19–22], although there are conflicting data regarding the association between HAE-C1INH and the risk of developing AD in general [14, 23–26]. The prevalence of ADs in patients with HAE is 12% compared with 4.5% (2.7% for males and 6.4% for females) in the general population [26, 27].

The objective of the current systematic literature review was to examine the number and type of co-occurring ADs reported in patients with HAE-C1INH based on relevant published mentions.

## Methods

A protocol for the systematic literature review was developed and registered in PROSPERO (CRD42018096855).

### Data sources and search strategy

Databases searched were PubMed/MEDLINE and EMBASE. PubMed/MEDLINE searches were limited to English publications up to the date April 15, 2018. EMBASE searches were limited to English abstracts from April 15, 2015 through April 15, 2018. Search strings based on key words or MeSH terms were used. The key word search string was “C1 AND inhibitor AND angioedema” and the MeSH term search string was “Complement C1 Inhibitor Protein”[MeSH]) AND “Angioedema”[MeSH]”. Search terms specific to ADs were intentionally not included in the search string since such a large number of ADs exist and it would not be possible to capture all potential ADs. When necessary to address the secondary objectives of the review, non-systematic PubMed searches were conducted to gather general information regarding genetics, etiology, and pathogenesis of HAE and ADs.

### Eligibility criteria

Review articles, case reports, observational studies, retrospective studies, and randomized controlled trials written in English in manuscript or congress abstract form were eligible for inclusion. Only published records that mentioned an AD in association with HAE Type I and II were included. Acquired angioedema (i.e., secondary to lymphoproliferative disorders) is also known to be associated with ADs but was excluded because the focus of this literature review was HAE-C1INH. Additional exclusions were HAE with normal C1-INH (HAE Type III), acquired angioedema associated with angiotensin converting enzyme inhibitors, non-human studies, and in vitro studies.

### Record selection and data collection processes

Titles and abstracts of records identified in the PubMed/MEDLINE and EMBASE searches were reviewed (in duplicate) by two reviewers to identify records of potential importance and, where discrepancies existed as to selected records, resolution was facilitated by a third reviewer. Full-length copies of the preliminarily identified manuscripts were obtained and reviewed by two reviewers within the context of the inclusion and exclusion criteria and reasons for any study exclusions were documented. Any discrepancies at the conclusion of the initial review process were resolved with the assistance of a third reviewer.

Records meeting the inclusion criteria and none of the exclusion criteria were reviewed by one reviewer and relevant information and data were extracted. Data were compiled into tables in an Excel format. A second reviewer conducted a quality assurance check with a duplicate review of approximately 10% of the records. When a record mentioned more than one patient with comorbid HAE-C1INH and AD, data from each patient was extracted individually and counted as a single mention. ADs extracted from the records were classified by MedDRA v21.0 High Level Terms (HLT).

The systematic literature review was intended to be exploratory and hypothesis generating. Furthermore, a broad scope of literature beyond clinical trials was assessed and risk of bias was not assessed.

## Results

**Fig. 1:**
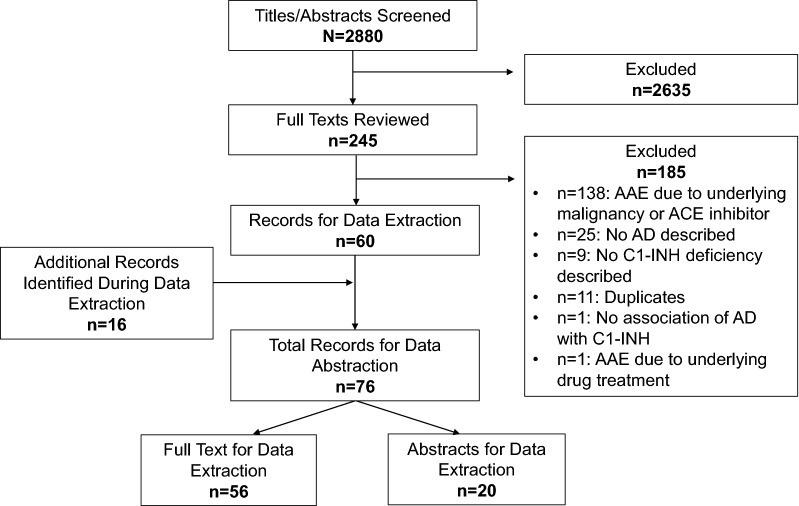
Systematic literature review record selection. *AAE* acquired angioedema, *ACE* angiotensin converting enzyme, *AD* autoimmune disease, *C1-INH* C1 esterase inhibitor

In all, 2880 titles and abstracts were screened and 245 were selected for full-text review. After full-text review, a total of 76 records were eligible for data extraction (Fig. 1). A total of 56/76 records were published as full-text manuscripts and 20/76 records were congress abstracts only. The majority of the 76 records (n = 48; 67%) were case reports or case series.

From the 76 records, 155 individual occurrences of HAE-C1INH and AD comorbidity were mentioned. Of these 155 co-occurrences, the most commonly identified ADs were SLE, thyroid disease, GN, rheumatoid arthritis (RA), Crohn’s disease, Sjogren’s syndrome, and celiac disease (Table 1). SLE was by far the most common co-occurring AD, accounting for 30 of the 155 reported occurrences. When the AD occurrences were grouped by MedDRA terms, groups with classified conditions of 5 or more occurrences were Lupus Erythematosus and Associated Conditions (n = 52), Endocrine Autoimmune Disorders (n = 21), Glomerulonephritis and Nephrotic Syndromes (n = 16), Gastrointestinal Inflammatory Conditions (n = 16), Rheumatoid Arthritis and Associated Conditions (n = 11), Eye, Salivary Gland and Connective Tissue Disorders (n = 10), and Immune and Associated Conditions Not Elsewhere Classified (n = 5; Table 2).

**Table 1:** Number of individual published mentions of HAE-C1INH and autoimmune disease comorbidity

Autoimmune disease	Number of HAE comorbid mentions
Lupus or lupus-like diseases	52
Systemic lupus erythematosus	30
Lupus (unspecified)	7
Discoid lupus	8
Lupus-like disease	4
Cutaneous lupus	2
Drug-induced lupus	1
Thyroid disease	21
Hypothyroidism	10
Thyroiditis	5
Anti-thyroid antibodies	3
Hashimotos	2
Unspecified	1
Glomerulonephritis	16
Rheumatoid arthritis	11
Crohn’s disease	8
Various autoantibodies	8
Sjogren’s syndrome	7
Celiac disease	7
Psoriasis	3
Antiphospholipid syndrome	3
Autoimmune hemolytic anemia/anemia	3
General/unspecified autoimmune disease	2
Psoriatic arthritis	2
Systemic sclerosis	2
Mixed connective tissue disease	2
Raynauds syndrome	2
Urticarial vasculitis	1
Sicca syndrome	1
Lipodystrophy	1
Alopecia	1
Multiple-sclerosis-like syndrome	1
Ulcerative colitis	1

*C1-INH* C1 esterase inhibitor, *HAE* hereditary angioedema

**Table 2:** Number of individual published mentions of HAE-C1INH and AD comorbidity grouped by MedDRA v21.0 high level terms

High level MedDRA term	Number of HAE comorbid AD mentions
Lupus erythematosus and associated conditions	*52*
Systemic lupus erythematosus	30
Lupus	7
Discoid lupus	8
Lupus-like disease	4
Cutaneous lupus	2
Drug-induced lupus	1
Endocrine autoimmune disorders	*21*
Thyroid diseases	21
Glomerulonephritis and nephrotic syndrome	*16*
Glomerulonephritis	16
Gastrointestinal inflammatory conditions	*16*
Crohn’s disease	8
Celiac disease	7
Ulcerative colitis	1
Rheumatoid arthritis and associated conditions	*11*
Rheumatoid arthritis	11
Eye, salivary gland and connective tissue disorders	*10*
Sjogren’s syndrome	7
Sicca syndrome	1
Mixed connective tissue disease	2
Immune and associated conditions not elsewhere classified	*5*
Psoriasis	3
Psoriatic arthritis	2

Groups with ≥ 5 mentions are shown

*AD* autoimmune disease, *C1-INH* C1 esterase inhibitor, *HAE* hereditary angioedema, *MedDRA* Medical Dictionary for Regulatory Activities

## Discussion

The results of the systematic literature review reveal a demonstrable co-occurrence of ADs in patients with HAE-C1INH, confirmed by clinical and biomarker evidence. Based on data collected in our systematic literature review, the most common co-occurring AD is SLE or lupus-like disease, followed by thyroid disease, GN, gastrointestinal diseases, RA, and Sjogren’s disease.

**Fig. 2:**
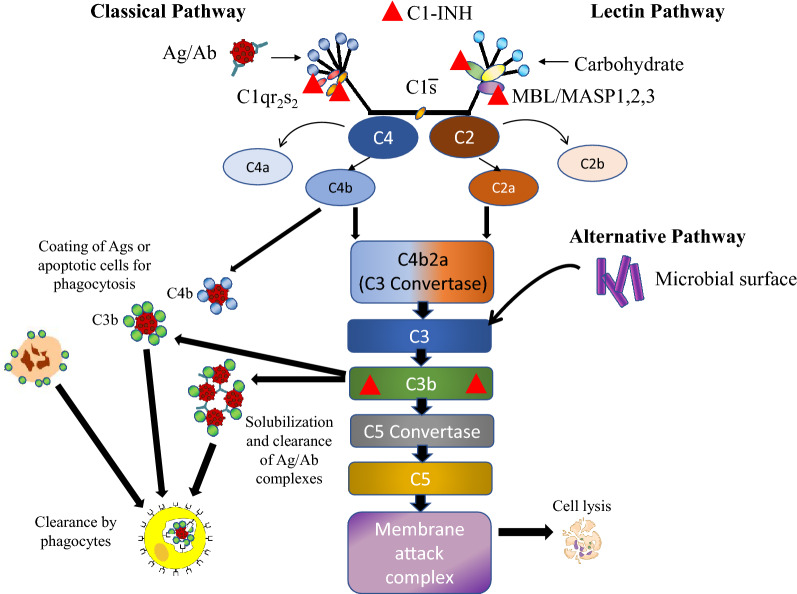
Complement mechanisms potentially protective against development of autoimmune disease. *Ab* antibody, *Ag* antigen, *C1-INH* C1-esterase inhibitor, *MASP* mannose-binding lectin-associated serine protease, *MBL* mannose-binding lectin

Although the C1-INH protein inhibits the development of autoimmunity by inhibiting both the classical and lectin complement pathways, the cause and effect between C1-INH deficiency (HAE) and development of ADs has yet to be established, and other etiologies need to be explored further. The lectin pathway has an activation scheme similar to that of the classical complement pathway, but lectins substitute for antibodies, and lectin-associated proteases replace C1r and C1s. The lectins bind sugar residues on microbial surfaces. MASPs subsequently cleave C4 and C2. C1-INH blocks the active sites of these MASPs (Fig. 2).

The alternative complement pathway generates a C3 convertase independent of C4 and C2 (C3bBb). Furthermore, C3 can spontaneously hydrolyze into C3a and C3b [28, 29]. Therefore, active C3b in patients with HAE-C1INH can still be produced to perform normal C3b functions, as evidenced by the low levels of circulating immune complexes in many patients with HAE-C1INH [19].

Reduced C4 levels as observed in SLE may be due to consumption or genetic deficiency of C4 alleles and both causes may be present in a given patient. In SLE, the measurement of C3 and C4 is typically used to assist the diagnosis and is useful for monitoring disease activity. SLE is also the prototypic disease for which the clinical information is available relative to interpreting and following low C3 and C4 levels. Low levels in SLE typically improve with treatment indicating classical pathway activation. Normalization of these complement values is also considered a good prognostic sign [30].

**Fig. 3:**
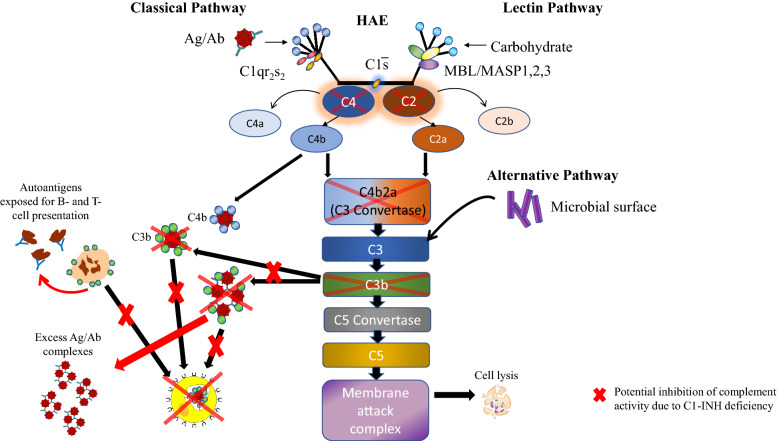
Potential role of C1-INH deficiency in development of autoimmune disease. *Ab* antibody, *Ag* antigen, *C1-INH* C1-esterase inhibitor, *HAE* hereditary angioedema, *MASP* mannose-binding lectin-associated serine protease, *MBL* mannose-binding lectin

Serum C4 is also typically low in patients with HAE because of chronic activation and depletion, whereas C2 and C3 may be low or normal; C2 levels decline during acute HAE attacks [24, 31–33]. In a retrospective observational study, patients with homozygous C4A deficiency were significantly more likely to have autoantibodies, SLE, and celiac disease compared with healthy controls [34]. Notably, mutations in the genes encoding C2, C3, C4A, C4B have been linked to SLE [35–37], and Sjogren’s syndrome has been reported in patients with genetic deficiencies in C1q, C4, and C2 [38–40]. Furthermore, the odds of having a C4B deficiency are two-fold higher in patients with RA vs non-RA patients [41]. Since subcomponents of C4 and C2 comprise the C3b convertase, which in turn acts upon C2 (Fig. 2), deficiencies of C2, C3, and C4 could result in a lack of C3b. C3b coats immune complexes and binds to complement receptor CR1, which then are opsonized and cleared by phagocytes (Fig. 2) [29, 42]. C3b also aids in the clearance of apoptotic cells by facilitating interaction between the apoptotic cell and phagocytes via binding of complement receptor CR3 [29]. Thus, C1-INH deficiency impacts the classical and lectin pathways and may predispose patients to increases in potentially damaging immune complexes (Fig. 3) [11, 17]. In addition, the defective clearance of apoptotic cells provides a source of autoantigens that can result in production of autoantibodies (Fig. 3) [11, 17]. Autoantibodies and deposition of immune complexes into tissues have been clearly linked to the pathogenesis of several of the ADs identified in this systematic review as most commonly co-occurring with HAE-C1INH, including SLE, GN, thyroid disease, RA, and Sjogren’s syndrome [18, 43–46]. Together these biological pathways indicate that the C1-INH protein may play a key role in the complement dysregulation by preventing excessive complement activation on a target, as well as in plasma (classical and lectin pathways). The decreased function of C1-INH with subsequent dysregulation of the classical and lectin complement pathways may lead to increased co-occurrence of AD in patients with HAE-C1INH. Certainly other factors other than complement dysregulation contribute to AD development. Genetic susceptibilities and triggers such as viral infection are also thought to be required to induce SLE, autoimmune thyroid disease, RA, and Sjogren’s syndrome [47–49].

Patients with SLE and other autoimmune conditions could develop acquired angioedema which can present with the same symptoms as HAE Type I or II along with a low C4 and a low C1 inhibitor function [50–52]. There would be no family history and the patients present with the angioedema as the first manifestation of their autoimmune condition. The only way to differentiate the problem is to do a C1q level, which may be low, or genetic testing. There is one case in the literature of a patient who had HAE Type I but developed features of acquired angioedema after she developed lymphoma [53]. Her C1q, which had been normal, fell and only recovered once her lymphoma was treated.

Prophylactic administration of C1-INH in HAE patients results in normalization or near-normalization of C4 and C1-INH antigen and functional levels, as evidenced in clinical trials and pharmacokinetic/pharmacodynamic modeling [54–56]. In a phase III clinical trial, the use of subcutaneous C1-INH resulted in sustained steady-state levels of functional C1-INH [54, 55]. It is unknown if the restoration of C4 levels in HAE patients treated with C1-INH prophylaxis affects any of the complement components or if there could be any additional benefits due to the potential restoration of proper complement regulation.

This systematic review is limited to search terms contained in the article’s title, abstract, and key words that mentioned the co-occurring HAE-C1INH and ADs, as described in the methods section. The prevalence of co-occurring HAE-C1INH and ADs cannot be inferred from this systematic review since the results only provide a report of the number of published mentions.

## Conclusions

Although there is a compelling association for the cause and effect of the co-occurrence of HAE-C1INH and AD due to persistently low C1-INH functional levels, more research is needed in HAE patients to confirm this theory. Further clinical observation and research is needed to see if subcutaneous C1-INH replacement therapy can possibly ameliorate or prevent AD in HAE patients.

## Data Availability

The datasets used and/or analysed during the current study are available from the corresponding author on reasonable request.

## Abbreviations

AD: Autoimmune disease
C1-INH: C1 esterase inhibitor
GN: Glomerulonephritis
HAE: Hereditary angioedema
MASP: Mannose-binding lectin-associated serine protease
RA: Rheumatoid arthritis
SERPING1: Serine protease inhibitor gene 1 present on the long arm of chromosome 11
SLE: Systemic lupus erythematosus
